# The Contribution of Allergen-Specific IgG to the Development of Th2-Mediated Airway Inflammation

**DOI:** 10.1155/2012/236075

**Published:** 2012-10-21

**Authors:** Jesse W. Williams, Melissa Y. Tjota, Anne I. Sperling

**Affiliations:** ^1^Committee on Molecular Pathology and Molecular Medicine, University of Chicago, Chicago, IL 60637, USA; ^2^Interdisciplinary Scientist Training Program and Committee on Immunology, University of Chicago, Chicago, IL 60637, USA; ^3^Section of Pulmonary and Critical Care Medicine, Department of Medicine, University of Chicago, Chicago, IL 60637, USA

## Abstract

In both human asthmatics and animal models of allergy, allergen-specific IgG can contribute to Th2-mediated allergic inflammation. Mouse models have elucidated an important role for IgG and Fc-gamma receptor (Fc**γ**R) signaling on antigen presenting cells (APC) for the induction of airway inflammation. These studies suggest a positive feedback loop between IgG produced by the adaptive B cell response and Fc**γ**R signaling on innate immune cells. Studies of IgG and Fc**γ**Rs in humans with asthma or allergic lung disease have been more controversial. Some reports have identified associations between allergen-specific IgG and severity of allergic responses, while other studies have found associations of IgG subclass IgG4 with allergic tolerance. In this paper, we review the literature to help define the nature of IgG and Fc**γ**R signaling on innate immune cells and how it contributes to the development of allergic immune responses.

## 1. Atopic Asthma Is Commonly Associated with Th2 Responses

Asthma is a chronic inflammatory disease of the lungs marked by recurrent episodes of airway hyperresponsiveness resulting in chest tightness, wheezing, and shortness of breath. Allergic or atopic asthma is the most common form of asthma, and allergic sensitization occurs in about 80% of asthmatic children and 60% of asthmatic adults [1]. Although there are now multiple phenotypes for atopic asthma, it has been classically associated with an excessive Th2-driven inflammatory response [2]. Development of an aberrant Th2 response leads to production of several cytokines including IL-4, IL-5, IL-9, and IL-13 that results in eosinophilia, goblet cell hyperplasia, mast cells activation, and smooth muscle hypertrophy [3]. In addition to these cellular effects, there is an important humoral response generated during primary sensitization that leads to production of allergen-specific IgE and IgG1. Much of the interest in dissecting the pathogenesis of asthma has focused on allergen-specific IgE which is well known to induce allergic hypersensitivity [4]. However, it was found that IgE^−/−^ mice were still able to develop anaphylaxis and airway hyperreactivity suggesting that other mediators including allergen-specific IgG could be playing an important role in disease pathogenesis [5, 6]. Moreover, the total allergen-specific IgG response is greater in magnitude and has a significantly increased half-life compared to the total allergen-specific IgE response [7]. The complex nature of IgE in promoting allergy can be reviewed in a variety of recent articles [8–10]. In this review, we discuss current research on IgG, Fc*γ*Rs, and allergy in order to better identify the role of IgG during allergic airway inflammation.

**Figure 1:**
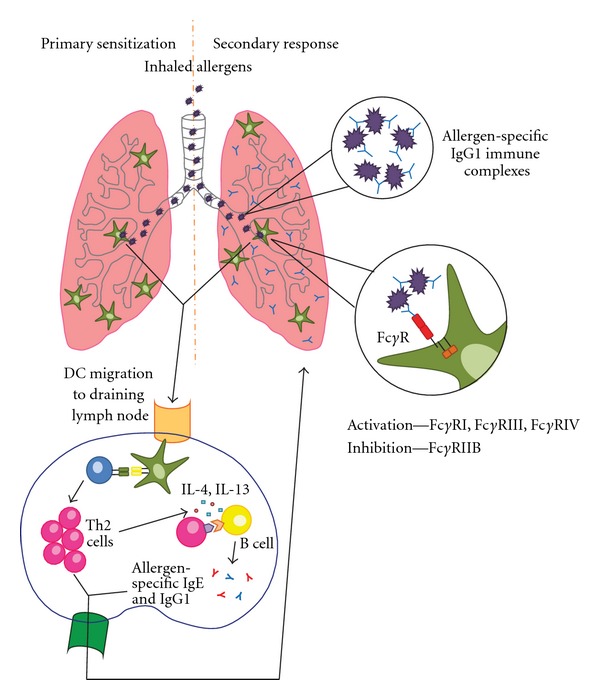
Model of IgG-mediated DC activation during secondary allergic responses in the lung. During primary sensitization, inhaled allergens are taken up by dendritic cells that then migrate to the draining lymph node to promote differentiation of T cells. Skewing towards a Th2 phenotype results in production of IL-4 and IL-13 that can promote IgE and IgG1 class switching in B cells. The IgG1 can complex with inhaled allergen during a secondary exposure to form ICs that signal through Fc*γ*Rs on hematopoietic cells to promote allergic responses.

Based on research discussed in this paper, we propose a model whereby allergen-specific IgG promotes the expansion of secondary Th2 responses through ligation of Fc*γ*Rs on innate immune cells (Figure 1). Allergen-specific IgG can be detected in the airways of sensitized individuals, and we propose that during secondary exposure to inhaled allergens, immune complexes (ICs) consisting of antigen and allergen-specific IgG are formed. These ICs can interact with both activating and inhibitory Fc*γ*Rs on innate immune cells and affect their activation and function. In our model, crosstalk between the IgG generated by the adaptive immune system and ligation of Fc*γ*Rs on innate immune cells can contribute to the pathogenesis of Th2 inflammation during secondary responses to inhaled allergens.

## 2. Fc**γ**R Expression and Function on Hematopoietic Cells

**Figure 2:**
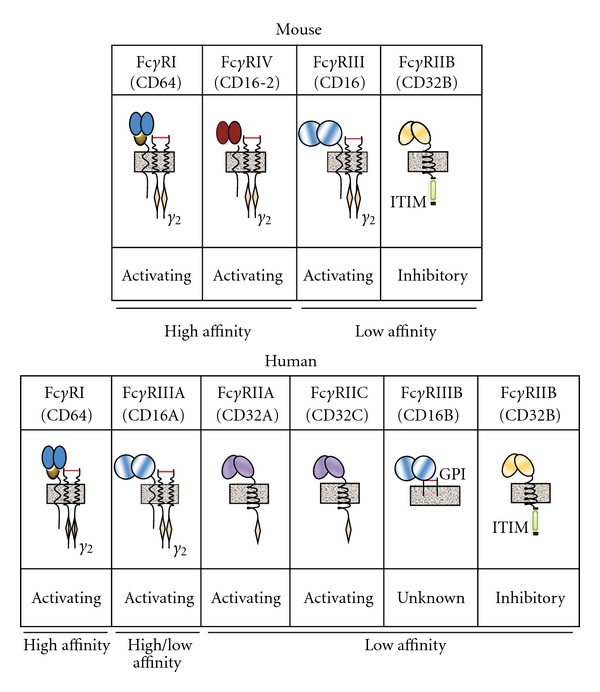
Mouse and human Fc*γ*R structure diagram. Receptors are labeled as “activating,” “inhibitory,” or “unknown.” Relative affinity of each receptor to monomeric (free) IgG is listed below each receptor [12, 16]. Signaling chains associated with each receptor are labeled; beige diamonds represent ITAMs, green box represents ITIM, and grey box represents cell membrane.

Fc*γ*Rs have emerged as an important bridge between the innate and adaptive arms of the immune system as they are primarily expressed on innate cells and their function can be affected by IgG ligation of Fc*γ*Rs. Thus far, there have been four Fc*γ*Rs identified in mice: Fc*γ*RI (CD64), Fc*γ*RIII (CD16), Fc*γ*RIV (CD16-2), and Fc*γ*RIIb (CD32) [11]. The family of Fc*γ*Rs is more complex in humans because multiple isoforms exist: Fc*γ*RI, Fc*γ*RIIA, Fc*γ*RIIB, Fc*γ*RIIC, Fc*γ*RIIIA, and Fc*γ*RIIIB [12]. In mice, Fc*γ*RI, Fc*γ*RIII, and Fc*γ*RIV are activating receptors that signal primarily through the Fc common *γ*-chain (FcR*γ*, noted as *γ*_2_ in Figure 2), and in humans, Fc*γ*RI, Fc*γ*RIIA, Fc*γ*RIIC, and Fc*γ*RIIIA are activating Fc receptors although only Fc*γ*RI and Fc*γ*RIIIA use FcR*γ* [13]. FcR*γ* contains immunoreceptor tyrosine-based activation motifs (ITAMs) that when phosphorylated allow Syk kinases to dock and become activated [14]. In contrast to mice, human Fc*γ*RIIA and Fc*γ*RIIC contain an intracellular ITAM, and human Fc*γ*RIIIB is a GPI-linked receptor only expressed on human neutrophils [12]. On the other hand, Fc*γ*RIIB in both humans and mice is an inhibitory receptor with an immunoreceptor tyrosine-based inhibitory motif (ITIM) in its cytosolic portion that will recruit and activate the SH2-domain containing inositol 5′ phosphatase (SHIP) upon phosphorylation [15]. A schematic of both mouse and human receptors along with their signaling chains is shown in Figure 2.

In addition to the numerous Fc*γ*Rs, there are four IgG subclasses present in mice (IgG1, IgG2a, IgG2b, and IgG3) and humans (IgG1, IgG2, IgG3, and IgG4). Each Fc*γ*R has a varying affinity for the monomeric IgG subclasses. Fc*γ*RI in mice and Fc*γ*RI in humans have the highest affinity for IgG and can bind monomeric IgG2a in mice or IgG1 and IgG3 in humans [17, 18]. The other Fc*γ*Rs have a significantly lower affinity for IgG and primarily bind to IgG-ICs [16]. In mice, Fc*γ*RIIB and Fc*γ*RIII are able to bind IgG1, IgG2a, and IgG2b while Fc*γ*RIV has been shown to bind IgG2a, IgG2b, and IgE [19–22]. In humans, IgG1 and IgG3 can be found bound by all the Fc*γ*Rs; IgG2 binds to allelic variants of Fc*γ*RII and Fc*γ*RIII; IgG4 binds to Fc*γ*RI, the Fc*γ*RII family, and an allelic variant of Fc*γ*RIII [12]. In both mice and human, IgG1 and IgG2 are found in the highest abundance in the serum. Human IgG4 is found at very low concentrations and is the only IgG subtype unable to form immune complexes or activate complement through binding C1q [23, 24]. Differences between IgG subtype expression and their affinity for activating or inhibitory Fc*γ*Rs may be important mechanisms for the regulation of allergic disease.

**Figure 3:**
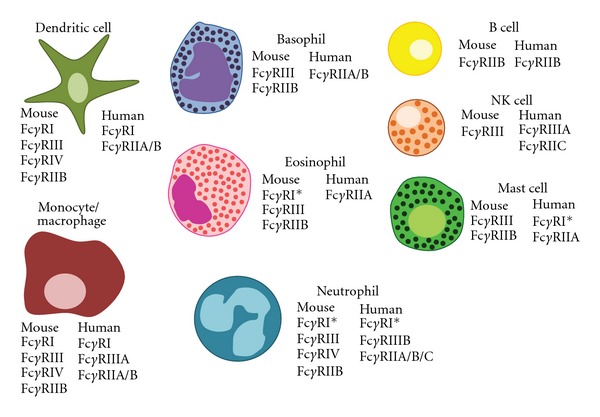
Human and murine Fc*γ*Rs are expressed on a variety of hematopoietic cells. Cells of the innate immune system express both activating and inhibitory Fc*γ*Rs: monocytes, macrophages, DCs, basophils, eosinophils, neutrophils, NK cells, and mast cells. On the other hand, B cells uniquely express Fc*γ*RIIb both in mice and humans. *denotes that expression can be induced upon activation.

Both activating and inhibitory Fc*γ*Rs can be expressed on hematopoietic cells, primarily innate immune cells, and the expression patterns of these receptors vary between mice and humans as highlighted in Figure 3. Most of the innate immune cells express both activating and inhibitory Fc*γ*Rs resulting in a certain threshold that will lead to the activation or inhibition of the immune response based on the ratio of these receptors and which IgG subclasses are present. This added level of control allows antibodies generated by the humoral immune system to play a critical role in affecting activation of innate immune cells through ligation of specific Fc*γ*Rs to induce immunogenic or tolerogenic responses, which will be discussed below.

## 3. Murine Models of Allergy Suggest an Important Role in IgG Signaling in the Development of Allergy

Preliminary studies investigating the contribution of Fc*γ*Rs in IgG-mediated disease models demonstrated that they helped regulate the immune response. The first knockout mouse model developed to address the role of IgG and activating Fc*γ*Rs in inflammatory responses was the FcR*γ* ^−/−^ mouse which had an attenuated Arthus reaction, an IC-mediated type III hypersensitivity response [25]. These results were confirmed in several other studies highlighting that FcR*γ* ^−/−^ mouse had significantly decreased IgG-mediated cellular responses [26–28]. However, the specific Fc*γ*Rs involved in mediating this response could not be identified because the FcR*γ* ^−/−^ mouse lacked all activating Fc*γ*Rs, so other studies developed specific Fc*γ*RI^−/−^, Fc*γ*RIII^−/−^, and Fc*γ*RIV^−/−^ mice to further clarify their role during in vivo immune responses. Studies in mice deficient for activating Fc*γ*Rs, particularly Fc*γ*RI and Fc*γ*RIII, showed decreased IgG-mediated responses in the knockout mice in several disease models: passive cutaneous anaphylaxis, complement-independent Arthus reactions, arthritis, IgG-dependent anaphylaxis, experimental autoimmune hemolytic anemia, bacterial infections, and glomerulonephritis [29–35]. Fc*γ*RIV is the most recently identified Fc*γ*R, and studies have demonstrated that it can contribute to IgG2b-mediated inflammatory responses in mice [36–40]. Recent studies of IgG1 and IgG2 signaling through Fc*γ*RIIIA and Fc*γ*RIV showed a neutrophil-dependent mechanism of anaphylaxis in both mice and humans [41]. The balance between activating and inhibitory Fc*γ*Rs has been shown to play a critical role in mediating inflammatory responses, so it was also important for investigators to analyze the role of the inhibitory receptor Fc*γ*RII. Early studies with the Fc*γ*RIIB^−/−^ mouse helped to confirm its role as an inhibitory inflammatory signal because these mice developed increased humoral and anaphylactic responses [42, 43]. Furthermore, it was found that Fc*γ*RIIB^−/−^ mouse developed increased Th2 responses in murine models of allergic airway inflammation [44–46]. Thus, the opposing effects of activating and inhibitory Fc*γ*Rs demonstrate the significant role that IgG and Fc*γ*Rs can play in modulating the immune response. Taken together, the results from the Fc*γ*R knockout mice indicate an ongoing interaction between the adaptive and innate immune system to shape the immune response.

The crosstalk between the humoral response and Fc*γ*Rs on innate immune cells suggests that allergen-specific IgG can contribute to the development and augmentation of Th2 responses in the lung during secondary responses to inhaled allergens. It was first seen that allergen-specific IgG could affect allergic airway inflammation on its own in the absence of a memory response utilizing passive transfer models. These studies highlighted that administration of antigen-specific IgG followed by antigen challenge could lead to the development of immediate hypersensitivity, airway hyperresponsiveness, and anaphylaxis [47, 48]. As outlined in Figure 1, formation of allergen-specific ICs could be one of the mechanisms by which allergen-specific IgG participates in the pathogenesis of allergic lung disease. This hypothesis is supported by studies investigating whether ICs alone could mediate Th2 responses in the lungs. It was shown that intranasal administration of anti-OVA IgG-ICs resulted in increased airway inflammation, eosinophilia, Th2 cytokine production, and antigen-specific T cell proliferation in an FcR*γ*-dependent manner [49]. In another study using airway hyperreactivity as a readout, mice were given polyclonal anti-BSA IgG intratracheally followed by an intravenous injection of BSA, and they developed severe airway hyperreactivity that peaked one hour after antigen administration and had resolved by 24 hours suggesting an immediate response to IgG-ICs [50]. These results indicate that allergen-specific IgG could exacerbate allergic lung diseases by forming allergen-specific IgG-ICs that promote activation of innate immune cells through interactions with Fc*γ*Rs.

Although several innate cells contribute to allergic lung disease, there has been a great deal of interest in understanding how DCs contribute to this process. It is well established that DCs are important in promoting Th2 inflammation in the lungs and can direct differentiation of CD4^+^ T cells into specific T cell lineages, and Fc*γ*Rs on DCs have been identified as being a potential mediator in affecting DC and Th2 responses in the lungs [49, 51–54]. Studies in our lab highlighted a role for Fc*γ*RIII in regulating Th2 responses; we demonstrated that when TLR4-stimulated DCs received an additional signal through Fc*γ*RIII, it led to augmented Th2 responses in murine models of allergic airway inflammation in an IL-10-dependent manner [53]. Our studies are supported by the finding that activation by IgG-ICs on macrophages and neutrophils results in heterodimerization of TLR4 and Fc*γ*RIII [55]. These conclusions suggest that IgG-ICs could provide a link between the adaptive and innate immune responses by modulating TLR signaling. Thus, a positive feedback loop between allergen-specific IgG and Fc*γ*Rs on DCs could drive established Th2 responses and exacerbate the development of allergic lung diseases. However, it should be noted that several studies have recently argued that free IgG1, not in immune complexes, and IVIg can bind Fc*γ*RIII and induce “inhibitory” ITAM signaling [56, 57]. The relevance of this effect may not be applicable to the development of allergic lung diseases because during secondary responses, ICs that bind to Fc*γ*RIII at a much higher affinity than free monomeric IgG are formed [16]. Other studies investigating the role of Fc*γ*RIIB on APCs have pointed to an inhibitory role for Fc*γ*R in Th2 responses. Sensitized wild-type mice receiving an intranasal aeroallergen challenge had an increase in Fc*γ*RIIB expression on respiratory CD14^+^/MHCII^+^ mononuclear cells and CD11c^+^ cells [44]. Furthermore, another study suggested that Fc*γ*RIIB on DCs contributed to tolerance induction against mucosal antigens [58]. One possible mechanism by which Fc*γ*RIIB on DCs ameliorates allergic airway inflammation is through inhibition of antigen uptake and DC activation [45]. Collectively, the studies in mice suggest that allergen-specific IgG generated during primary sensitization complexes with inhaled antigen during secondary responses and depending on the balance of Fc*γ*Rs signaled on DCs, can either positively or negatively affect the development of Th2 inflammation in the lungs.

## 4. Fc**γ**R and IgG in Human Allergy and Asthma

The complexity of the IgG subclasses and Fc*γ*Rs in humans has made it more difficult to determine the effect of allergen-specific IgG on the development of Th2 responses in allergic diseases. Most studies in humans have investigated the correlation between different IgG subclasses, their specificities, and association with allergic phenotypes, while only a few studies have investigated the role of IgG signaling and their receptors in human allergic disease. Correlations between atopy and Fc*γ*R expression levels have provided conflicting results yet overall suggest that increased expression of activating Fc*γ*Rs is augmented in allergic individuals [59–62]. Interestingly, one study investigating the ability of human Fc*γ*RIII^−^ and Fc*γ*RIII^+^ monocyte-derived DCs (mDCs) to reactivate memory responses found that Fc*γ*RIII^+^ mDCs stimulated stronger T cell responses in vitro [63]. In general, determining the function of human Fc*γ*Rs has proven difficult as there are limited reagents and variable populations being studied. Currently, studies have utilized transgenic mice overexpressing human Fc*γ*R genes to study function, but these studies are often limited by cell expression and binding affinity to mouse IgG. To examine the role of human Fc*γ*Rs in models of allergic disease, human Fc*γ*RIIA which binds mouse IgG1, IgG2a, and IgG2b was overexpressed in an Fc*γ*R^−/−^ mouse and assayed for anaphylactic responses. Expression of only human Fc*γ*RIIA in mice was sufficient to induce both active and passive anaphylaxis, as well as acute allergic responses [64]. Similarly, an Fc*γ*R humanized mouse that expresses the entire human Fc*γ*R family and lacks all mouse Fc*γ*Rs was generated. These mice were able to mount comparable immune responses to wild-type mice during immune-complex mediated anaphylaxis, NP-OVA sensitization and challenge, Fc*γ*RIIB-dependent vaccination, and antitumor immunity; thus, they could prove to be a valuable tool in clarifying the role of human Fc*γ*Rs [65]. While these studies utilized human Fc*γ*Rs expressed in mice, together they confirm the hypothesis that Fc*γ*Rs contribute to the development of allergic disease and demonstrate that human receptors display similar phenotypes to their murine counterparts.

Several studies have found a contributing role for antigen-specific IgG in the pathogenesis of asthma in humans. Notably in 2005, the German Multicentre Allergy Study published results from a longitudinal study of cat exposure and asthma development in children from age 6 months to 10 years. As expected this study found that cat Fel d 1 antigen-specific IgE levels were associated with increased asthma risk but surprisingly found that children with both antigen-specific IgE and IgG antibodies showed the greatest risk for asthma. This study and other studies did not find a correlation between allergen-specific IgG alone and increased asthma risk [66]. A similar study investigated childhood exposure and antibody responses to both house dust mites (HDM) and cats and determined that both total antigen-specific IgG and IgG4 to cat Fel d 1 and HDM Der f 1 paralleled the level of total antigen exposure. Analysis of these populations showed a strong association with HDM-specific IgG, but not cat-specific IgG, for increased risk of asthma [67]. These studies suggesting a role for allergen-specific IgG in the promotion of Th2-mediated allergic disease are further supported by data showing elevated levels of IgG1 and IgG4 in the bronchoalveolar lavage fluid (BALF) of individuals with asthma [68, 69]. Furthermore, the induction of IgG during allergic Th2 responses is not limited to asthma; in other Th2-mediated diseases individuals with allergic bronchopulmonary aspergillosis and allergic alveolitis (e.g., farmer's lung and bird fancier's disease) are associated with increased levels of antigen-specific IgG [70, 71]. Together, these studies point to an important association between allergic IgG response and the perpetuation of airway inflammation in allergic individuals and support our model of antigen-specific IgG influencing the innate response to promote allergy.

It has been proposed that in some contexts IgG may be able to block normal IgE antigen binding and thereby inhibit the allergic response. IgG-mediated tolerance induction is thought to result from an accumulation of IgG4 and loss of IgE responsiveness. One study investigating the role of IgG in HDM responses found that allergic children were strongly associated with increased HDM-specific IgG1 and IgG4 levels [72]. However, supporting an inhibitory role of IgG4 in human allergic responses, this study compared children needing hospitalization as a result of acute asthmatic exacerbation and found a dramatic loss of both antigen-specific IgG1 and IgG4 compared to nonhospitalized allergic children, while HDM-specific IgE remained similar in magnitude and specificity between groups [72]. These findings emphasize the relationship between the presence of IgG4 and controlling asthma in children, suggesting that IgG4 could be ameliorating asthmatic disease in children. The prevalent hypothesis for IgG4 in asthma is that antigen-specific IgG4 can block IgE binding on allergens to reduce hyperresponsiveness. To test the ability of IgG4 to block IgE binding to antigen, IgG4^+^ B cells were isolated from human patients undergoing allergen immunotherapy and successfully cloned IgG4 antibodies. One cloned IgG4 antibody was found to be specific against the grass pollen allergen Phl p 7, from *Phleum pratense,* and was shown to be able to modify IgE responses and inhibit basophil activation in vitro [73]. These results show that IgG4 in some context can block IgE binding to specific epitopes on antigen; however, the authors did not emphasize the low percentage of antigen-specific IgG4 found in their sample population [73]. A similar study analyzing Birch pollen IgG1 found similar inhibitory effects suggesting that IgG1 could be overlapping epitopes with IgE and may not be unique to IgG4 [74]. Additionally, it has also been shown that the diversity and affinity of the antigen-specific IgE response may be an important part in the activation of human Th2 responses and could explain in part why IgG4 may be able to block responses in some patients and not others [75]. These studies argue a possible role of IgG4 antibodies in displacing IgE binding to allergen epitopes and thereby reducing allergic susceptibility.

In contrast, multiple studies have found that elevated levels of IgG4 had no effects on allergic disease [76–78]. A recently published study investigating newly exposed laboratory-animal workers revealed that IgG4 levels did not change over two years of tracking and no correlation between newly sensitized IgE producing individuals to rodents and levels of antigen-specific serum IgG4 [77]. Similarly, school children who owned cats were associated with increased IgG4 levels but were not protected from developing asthma [76]. Moreover, allergic patients treated with grass pollen immunotherapy for two years had augmented antigen-specific IgG during therapy but returned to pretreatment IgG levels quickly following the end of therapy without a loss of tolerance [79]. Further, it is well known that IgG4 is unable to form immune complexes, activate complement, nor does it have a high affinity for Fc*γ*Rs. Together, these findings suggest that even though IgG4 is induced in response to constant antigen exposure, it may act as a bystander and not a bona fide mechanism for mediating allergic tolerance. Overall, data has suggested that IgG in some context may be capable of overlapping epitope binding with IgE responses in order to dampen allergic sensitivity; however, it appears that it may not be a common mechanism and more research will be needed to elucidate these contradicting results.

One mechanism of immune regulation that might reconcile the seemingly contradictory findings presented above is recent studies suggesting a role for regulatory T cell populations (Treg or Tr1) in the development of both tolerance and IgG4 following allergen-specific immunotherapy [80–82]. Treg-produced IL-10 may contribute to the effects of immunotherapy by enhancing the survival and proliferation of previously differentiated IgG4 B cells [81]. Additionally, IL-10 treatment of IL-4-treated peripheral blood mononuclear cells (PBMCs) can promote the production of IgG4 and decreased IgE antibodies from peripheral B cells [81]. Finally, purified IL-10-producing Tr1 cells or Treg cells co-cultured with PBMCs from allergic individuals can modulate the B cell response to HDM antigen Der p 1 away from IgE and towards IgG4 [82]. Together these human studies identify an important role for regulatory T cell subsets on the development of tolerance in allergy. Importantly, these results suggest that the correlation of elevated levels of IgG4 with tolerance during immunotherapy may actually be a side effect of increased IL-10 in the serum and not a direct effector mechanism.

## 5. Discussion

Although the mechanisms by which IgG contributes to the pathogenesis or tolerance of allergic responses remain controversial, we believe that the evidence from both mouse and human studies points towards an important role for IgG in the regulation of allergic phenotypes. Clinical studies have demonstrated that in addition to elevated levels of allergen-specific IgE, allergic individuals also have elevated levels of allergen-specific IgG. Our previously published results showed that the activating receptor Fc*γ*RIII was necessary for potent induction of Th2 responses [53]. Further we found that Fc*γ*RIII is able to modulate TLR signaling on DCs in order to drive Th2 responses [53]. Based on these results and the data presented above, we propose a model (Figure 1) whereby primary sensitization to an allergen results in B cell class switching and the production of allergen-specific IgG1. IgG1 can be found present in the lungs of sensitized individuals and will be able to complex with inhaled allergens during secondary responses. Allergen-ICs in the lung can signal through the activating Fc*γ*Rs on innate immune cells, in particular Fc*γ*RIII. We have found that signaling through this receptor induces DC that specifically augments Th2 development through the expression of multiple genes. These migratory respiratory DCs can provide a milieu supportive of allergic responses in the airways thereby providing a positive feedback loop between the development of B cells that produce allergen-specific IgG, and the innate response of the respiratory DCs in the lungs. While we propose a positive feedback of allergen-specific IgG, the expression levels and affinity of different Fc*γ*Rs on innate cells in the lungs may explain the differential results found between some studies. The presence of different Fc*γ*Rs and different types of innate cells in normal versus atopic individuals could determine whether antigen-specific IgG contributes to the development of allergy or tolerance. Specifically, inhibitory Fc*γ*R receptors may explain how in some murine studies Fc*γ*RIIB seems to be able to overcome the activating Fc*γ*Rs to ameliorate disease. It is known that Fc*γ*RIIB has lowered affinity to IgG compared to the activating receptors, therefore suggesting that results studying Fc*γ*RIIB in mice may not be the same in human allergy. Thus to thoroughly understand the importance of the IgG response in allergy, questions remain such as the differential expression of IgG subtypes, differences in IgG-affinity, and expression levels of Fc*γ*Rs on innate cells between allergic and nonallergic individuals.
